# Functional Analysis of *preA* in *Aeromonas veronii* TH0426 Reveals a Key Role in the Regulation of Virulence and Resistance to Oxidative Stress

**DOI:** 10.3390/ijms21010098

**Published:** 2019-12-21

**Authors:** Bintong Yang, Haichao Song, Dingjie An, Dongxing Zhang, Sayed Haidar Abbas Raza, Guiqin Wang, Xiaofeng Shan, Aidong Qian, Yuanhuan Kang, Chunfeng Wang

**Affiliations:** 1 College of Animal Science and Technology, Jilin Provincial Engineering Research Center of Animal Probiotics, Key Laboratory of Animal Production and Product Quality Safety of Ministry of Education, Jilin Agricultural University, Changchun 130118, China; gracebintong@163.com (B.Y.); shc19931229@163.com (H.S.); andingjie@126.com (D.A.); zhangdx1992@163.com (D.Z.); wgqjlau@alyun.com (G.W.); sxf1997@163.com (X.S.); qianaidong0115@163.com (A.Q.); 2 College of Life Science, Changchun Sci-Tech University, Shuangyang District, Changchun 130600, China; 3 College of Animal Science and Technology, Northwest A&F University, Yangling 712100, China; haiderraza110@nwafu.edu.cn

**Keywords:** *Aeromonas veronii*, *preA* gene, virulence, oxidative stress

## Abstract

*Aeromonas veronii* is one of the main pathogens causing freshwater fish sepsis and ulcer syndrome. This bacterium has caused serious economic losses in the aquaculture industry worldwide, and it has become an important zoonotic and aquatic agent. However, little is known about the molecular mechanism of pathogenesis of *A. veronii*. In this study, we first constructed an unmarked mutant strain (Δ*preA*) by generating an in-frame deletion of the *preA* gene, which encodes a periplasmic binding protein, to investigate its role in *A. veronii* TH0426. Our results showed that the motility and biofilm formation ability of Δ*preA* were similar to those of the wild-type strain. However, the adhesion and invasion ability in *epithelioma papulosum cyprini* (EPC) cells were significantly enhanced (2.0-fold). Furthermore, the median lethal dose (LD_50_) of Δ*preA* was 7.6-fold higher than that of the wild-type strain, which illustrates that the virulence of the mutant was significantly enhanced. This finding is also supported by the cytotoxicity test results, which showed that the toxicity of Δ*preA* to EPC cells was enhanced 1.3-fold relative to the wild type. Conversely, tolerance test results showed that oxidative stress resistance of Δ*preA* decreased 5.9-fold compared to with the wild-type strain. The results suggest that *preA* may negatively regulate the virulence of *A. veronii* TH0426 through the regulation of resistance to oxidative stress. These insights will help to further elucidate the function of *preA* and understand the pathogenesis of *A. veronii.*

## 1. Introduction

*Aeromonas veronii* is a Gram-negative facultative anaerobe that is widely distributed in nature, especially in freshwater environments, with strong environmental adaptability [1]. In recent years, a growing number of cases of large-scale *A. veronii* outbreaks have resulted in the infection of freshwater fish, livestock, birds, and red meat animals (such as camels). These events can cause severe losses to the breeding industry and threaten food hygiene and safety [2,3,4]. Moreover, *A. veronii* has been reported to be the etiological agent of gastrointestinal infections and a wide spectrum of extra-intestinal infections, including wound infection, septicemia, urinary tract and soft tissue infections, and septic arthritis in humans [5]. The virulence and antimicrobial resistance of clinically isolated strains are gradually increasing, and the dangers to the aquaculture industry and the threat to public health continue to grow. *A. veronii* has become an important pathogen of humans, and veterinary and aquatic animals [6,7]. Only a few decades have passed between the initial discovery of *A. veronii* and the explosive epidemic in many parts of the world; however, the research on this bacterium has been limited to the isolation and identification of certain diseased organisms and some related phenotypes, while little is known about its pathogenesis and virulence. Therefore, an in-depth study of the effects of virulence factors is particularly crucial for elucidating the pathogenesis of *A. veronii*.

The periplasmic binding protein encoded by the *preA* gene is a high-pH-induced lipid-binding protein and is present in various microorganisms [8,9]. The periplasmic protein, which was identified as *ygiT* in high-pH environments, regulates redox reaction with the product of a putative membrane-bound redox reaction modifier [10,11]. The proteins are involved in the biosynthesis of isoprenoid quinone, which is widely distributed among prokaryotes and eukaryotes. Related research has shown that isoprenoid quinones are essential components of the membrane-bound electron-transport system and play essential roles in respiratory electron transport, oxidative stress control, and gene regulation [12]. Sequence homologs of this protein are ubiquitous in bacteria and archaea, with a structure that consists of an expanded eight-parallel “barrel” [13]. The protein binds to polyisoprene chains through hydrophobic interactions in the hydrophobic pores of the barrel [14]. To date, the YceI-like family and all other homologs have been annotated as hypothetical proteins. Crystal structure studies have indicated that this family of proteins plays a crucial role in the metabolism, transport, and storage of isoprenoids [15]. Pathogenic bacteria can enhance their adaptability to the external environment by controlling the expression of the *preA* genes, including complex biologic processes such as accumulation of glutamic acid, synthesis of trehalose, and the release of putrescine [16], which is conducive to the enhancement of pathogens. However, the specific function of *preA* in *A. veronii* has not been studied.

In the present study, the main structure of the *preA* gene was deleted from the *A. veronii* genome by homologous recombination to generate the mutant ∆*preA*. An overexpression strain (complemented strain, C-*preA*) of the *preA* gene was constructed by a broad-host-range expression plasmid. We used various biological methods to investigate the function of the *preA* gene in *A. veronii*. This study represents the first exploration of the function of the *preA* gene. The results will provide theoretical support for elucidating the complex pathogenesis of *A. veronii* and lay a solid foundation for further solving the mystery surrounding this microorganism to develop vaccines and prevent and treat the disease caused by *A. veronii* infection in animals.

## 2. Results

### 2.1. Construction and Characterization of the Mutant Strain ∆preA and Complemented Strain C-preA

**Figure 1:**
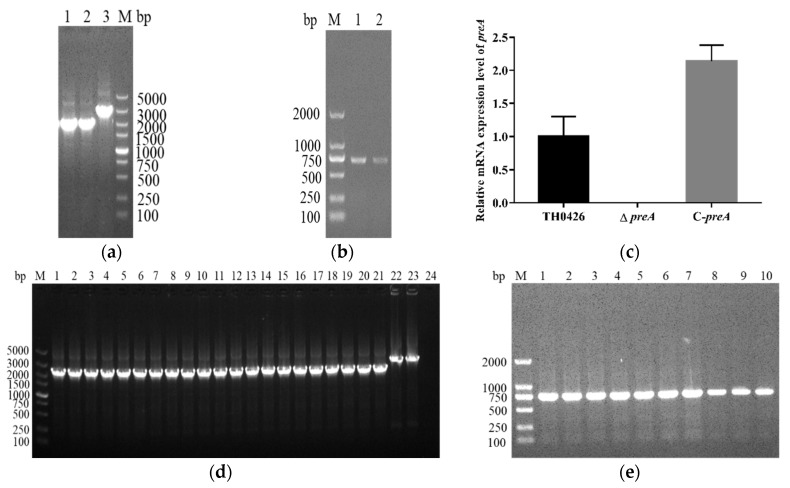
Construction and validation of mutant ∆*preA* and complemented strain C-*preA* of *Aeromonas veronii*. (**a**) PCR detection of deletion strain ∆*preA* (M: 5000 bp marker; 1, 2: the suspected mutant strain; 3: the wild-type strain of TH0426); (**b**) PCR verification of complemented strain C-*preA* (M: 2000 bp marker; 1: the wild-type strain; 2: C-*preA*); 2: C-*preA*; (**c**) analysis of the relative mRNA expression of the *preA* gene; (**d**) PCR analysis of the genetic stability of partial deletion strain (M: 5000 bp marker; 1–21: the mutant strain ∆*preA*; 22–23: the wild-type TH0426; 24: negative control); (**e**) genetic stability analysis of partial complemented strain C-*preA* (M: 2000 bp marker; 1–8: the complemented strain C-*preA*; 9–10: the parent strain).

The mutant strain ∆*preA* and complemented strain C-*preA* were generated by homologous recombination to examine the role of the *preA* gene in *A. veronii*. The ∆*preA* and C-*preA* strains were confirmed by PCR and qRT-PCR. The detection results of both strains showed that they were constructed successfully (Figure 1a,b). The qRT-PCR assay showed that the *preA* gene was expressed in both the wild-type and complemented strains, while no signal was present in the ∆*preA* strain (Figure 1c), which also confirmed the successful construction of the two variant strains. Furthermore, PCR analysis revealed that the altered genomes of both newly generated strains could be stably inherited for more than 50 generations (Figure 1d,e).

### 2.2. The preA Gene is not Involved in Growth Regulation

**Figure 2:**
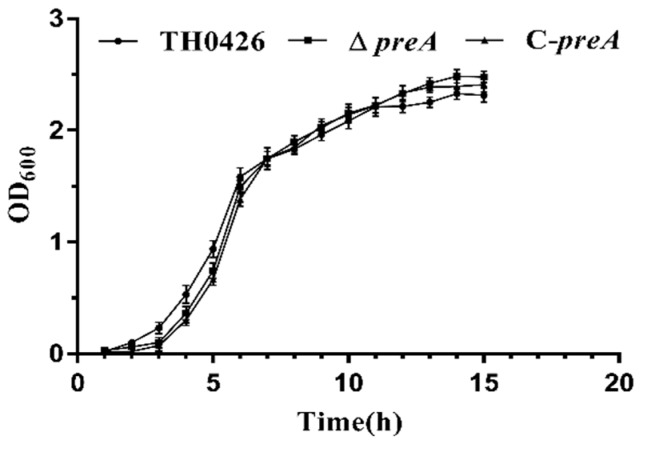
Growth curve measurement of *A. veronii* after *preA* deletion compared with the complemented (C-*preA*) and wild-type (TH0426) strains. Results are presented as means ± standard deviations (*n* = 3).

The growth curves of ∆*preA* and C-*preA* were constructed and compared with that of the wild-type strain TH0426 to determine the function of the *preA* gene during growth. Surprisingly, almost no difference in growth ability was observed between the mutant strain and the wild-type and complemented strains. There was a slight decrease in the logarithmic phase of growth, and the stability period increased slightly (Figure 2). However, the difference was not significant.

### 2.3. The Contribution of the preA Gene to Motility

**Figure 3:**
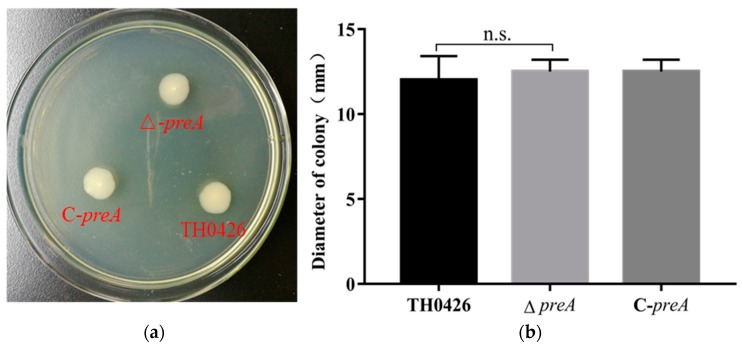
The impact of the *preA* gene on the motility of *A. veronii*. (**a**) *A. veronii* has swimming ability. (**b**) The swimming diameter was measured to reflect the change in motility (n.s., no significant difference).

Motility detection results indicated that wild-type *A. veronii* demonstrated swimming ability but lacked swarming ability, which is consistent with previous research results. The distances traveled by the parent strain, ∆*preA,* and C-*preA* were measured, as shown in Figure 3. The results showed no significant differences between the three strains.

### 2.4. preA Mutation does not Change the Biofilm Formation Ability of TH0426

**Figure 4:**
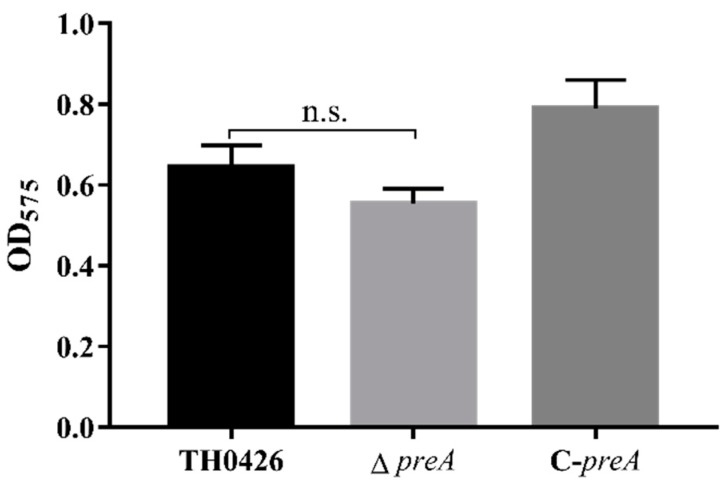
Biofilm formation ability of the wild-typ, ∆*preA*, and C-*preA* strains of *A. veronii*. The amount of biofilm formation is represented by the OD_575_ value (n.s., no significant difference).

According to previous reference standards of biofilm formation capability, an OD_575_ value that is less than or equal to the value for the control group indicates a lack of biofilm formation ability. Weaker, moderate, and stronger biofilm formation ability are indicated according to OD_575N_ < OD_575_ ≤ 2OD_575N_; 2OD_575N_ < OD_575_ ≤ 4OD_575N_; and OD_575_ > 4OD_575N_, respectively. *A. veronii* TH0426 was identified as having moderate biofilm formation capacity. The test results showed that the biofilm formation ability of the mutant Δ*preA* was decreased by the inactivation of the *preA* gene (Figure 4), but the difference was not significant, indicating that the *preA* gene does not regulate the biofilm formation of *A. veronii*.

### 2.5. LD_50_ and Virulence

**Figure 5:**
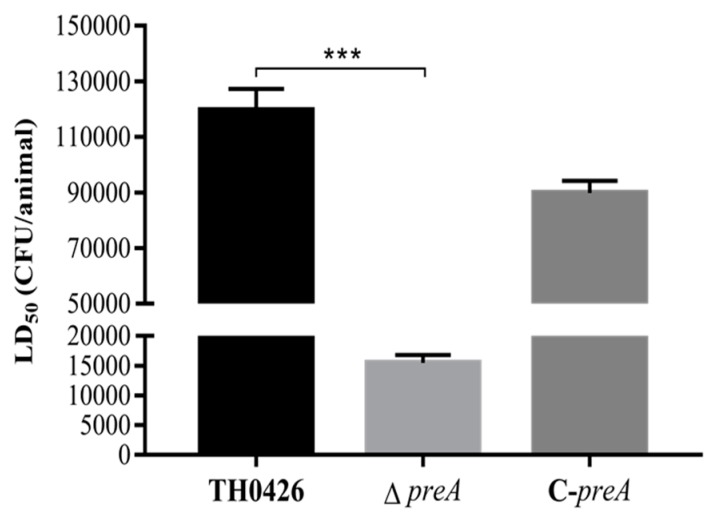
The effect of *preA* on virulence in adult zebrafish. The survival rates of zebrafish challenged by wild-type TH0426, ∆*preA*, and C-*preA* (*** *p* < 0.001, significant difference).

The role of the *preA* gene in the virulence of *A. veronii* TH0426 was assessed in vivo and in vitro. The median lethal doses (LD_50_) of the three strains (wild-type TH0426, ∆*preA*, and C-*preA)* in zebrafish were (1.25 ± 0.15) × 10^5^ CFU/fish, (1.64 ± 0.21) × 10^4^ CFU/fish, and (8.68 ± 0.18) × 10^4^ CFU/fish, respectively. The results showed that the LD_50_ of the mutant strain ∆*preA* was 7.6-fold lower than that of the wild-type strain TH0426, and the virulence was significantly enhanced (*p* < 0.001) (Figure 5). The virulence of C-*preA* was slightly restored, but the change was not significant.

The results of cytotoxicity tests showed that there was no significant change between the strains within 30 min of infection. As shown in Table 1, the changes began to appear 1 h after infection. The virulence of the mutant Δ*preA* in epithelioma papulosum cyprini (EPC) cells was 1.4-fold higher than that of the wild-type TH0426 strain, which indicates significantly enhanced virulence of Δ*preA*. At 2 h post-infection, the virulence of the deletion strain ∆*preA* in EPC cells was 1.3-fold higher than that of the parent strain; the difference between the two strains was extremely significant (*p* < 0.01).

### 2.6. The Adhesion and Invasion Ability against EPC Cells

Table 2 compares the capacities for adhesion to EPC cells of the parent and the other two strains (mutant and complemented strains). Adhesion is expressed as the ratio of final colony count to the initial infection bacterial count. The adhesion and invasion abilities of the mutant ∆*preA* to EPC cells were 33% ± 0.3%, and that of the wild type was 16% ± 1.1%. The difference between the two strains was significant; thus, deleting the *preA* gene in *A. veronii* significantly enhanced its cell adhesion and invasion ability. C-*preA* recovered slightly but did not reach the level of the wild-type strain.

### 2.7. The Role of preA in Environmental Stress of A. Veronii

**Figure 6:**
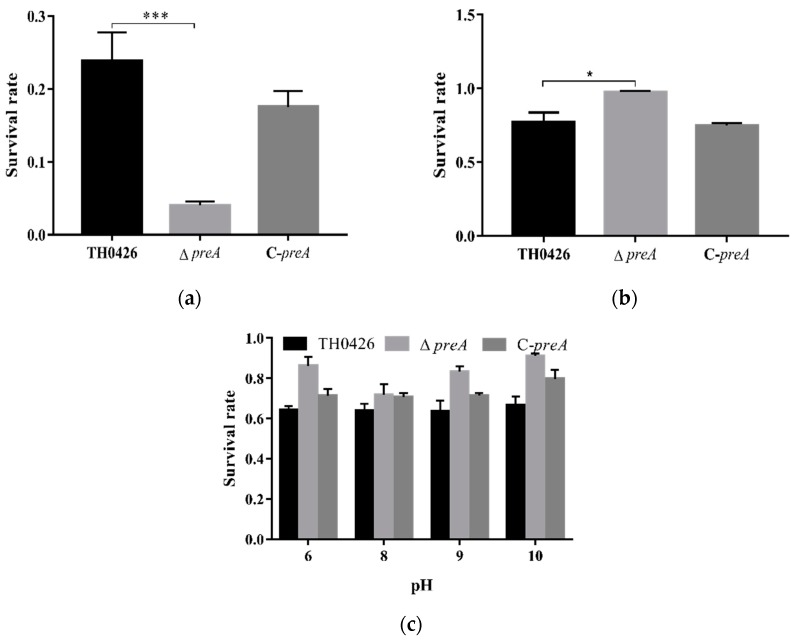
Tolerance of *A. veronii* to various environmental conditions. (**a**) Survival rate of the wild-type TH0426, Δ*preA* mutant, and complemented strains after oxidative stress (H_2_O_2_) treatment; (**b**) osmotic tolerance of the three strains; (**c**) tolerance of each strain to different pH values. * *p* < 0.05, *** *p* < 0.001 compared with the wild-type strain (TH0426).

The number of surviving bacteria was determined using plate counting method. The three strains did not survive under high-temperature conditions (data not shown), but they showed tolerance to high osmotic pressure and pH (Figure 6b,c). After exposure to H_2_O_2_, the survival rates of the parent, *preA* deletion mutant, and complemented strains were 26.6%, 4.4%, and 19.1%. Oxidative stress resistance decreased significantly (*p* < 0.001) in the deletion strain Δ*preA* compared with the parent strain (Figure 6a). These results indicate that the *preA* gene plays a key role in the resistance to oxidative stress in *A. veronii*.

### 2.8. Variation in the Expression of Oxidative Stress-Related Genes

**Figure 7:**
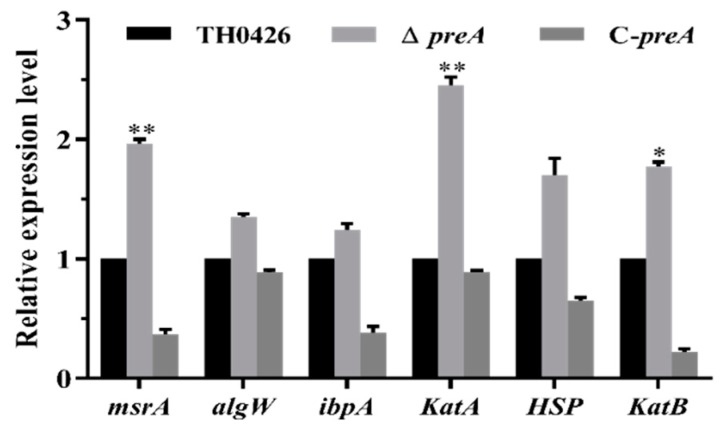
Expression changes of oxidative stress-related genes in the wild-type TH0426, Δ*preA* mutant, and complemented strains of *A. veronii*. * *p* < 0.05, ** *p* < 0.01 compared with the wild-type strain (TH0426).

The tolerance test results showed that the oxidative stress tolerance of *A. veronii* was significantly reduced by the deletion of the *preA* gene. Therefore, we examined the expression of several oxidative stress-related genes to further investigate the changes in oxidative stress tolerance. The results showed that the tolerance-related genes in the metabolic pathway of the periplasmic binding protein were significantly up-regulated, and no down-regulated genes were found (Figure 7).

## 3. Discussion

The virulence of *A. veronii* has been significantly increasing since its discovery, and the incidence of infection by this pathogen is showing an upward trend. The pathogenic mechanism of *A. veronii* is extremely complicated, and its tolerance to external environmental stress determines the ability of the strain to stably exert virulence. Tolerance to external stress requires metabolic adjustments by the bacteria to suit the environment. The YceI protein family is widely distributed among Gram-negative bacteria [17] and includes periplasmic binding proteins that play important roles in the metabolism, transport, and storage of isoprenoids [15]. The high-pH-induced lipid-binding protein encoded by the *preA* gene is reported to be a member of the YceI protein family, which is involved in the transportation of virulence proteins in pathogens and has a significant role in oxidative stress and gene regulation [12]. The expression of the *preA* gene is greatly affected by the external environment: pathogenic bacteria can enhance their adaptability to their surroundings by controlling the expression of *preA* [16], which lays a foundation for the pathogenicity of infectious microbes.

The earliest report on the YceI protein family was produced when F. R. Blattner sequenced the whole genome of *Escherichia coli* K-12 strain for the first time [18]. The functions of YceI protein family members were first reported in *E. coli,* and they were described as important modifiers of redox reactions and capable of binding to polyisoprene. In addition, these proteins play important roles in respiratory electron transport, oxidative stress control, and gene regulation. To date, proteins with high homology to the periplasmic protein encoded by the *preA* gene have been reported only in common intestinal pathogens, such as *Escherichia coli* and *Salmonella*. Interestingly, the protein database shows that proteins in *Aeromonas* that share more than 80% homology with *preA*-encoded protein are almost all uncharacterized or hypothetical proteins. In *A. veronii,* the protein is uncharacterized. However, the related functions of the *preA* gene in *A. veronii* have not been reported.

In this experiment, the mutant strain Δ*preA* of *A. veronii* TH0426 was successfully constructed by deleting the *preA* gene, and the complemented strain C-*preA* of Δ*preA* was simultaneously constructed using the broad-host-range expression plasmid pBBR1-MCS. Biological characterization revealed that the growth characteristics and biofilm formation ability of the mutant strain Δ*preA* did not differ from those of the wild-type strain TH0426. In tolerance tests at different pH values, the survival rates of the mutant strain ∆*preA* were 83.1%, 75.9%, 81.3%, and 92.1% at pH 6, 8, 9, and 10, respectively. There was no significant difference in pH tolerance compared with the parent strain TH0426, indicating that the deletion of the *preA* gene would not fundamentally affect the survival of *A. veronii* in high-pH environments. However, this finding is contrary to the results of Lauren M. Stancik et al. [19], who studied the pH-dependent expression profiles of periplasmic protein family genes in *E. coli*. They found that the expression of the YceI protein family gene was heavily dependent on pH changes and indicated the need for further research to clarify the complex phenomenon. In the present study, there was no significant difference in the survival rate between the three strains under high-temperature stress (data not shown), but changes in osmotic stress and exposure to a strong oxidative environment revealed a significant difference in the survival of the deletion strain ∆*preA*. Compared with the wild-type strain, the survival rate of ∆*preA* was significantly higher in a hypertonic environment, whereas the antioxidative capacity of ∆*preA* was significantly lower. These results differ from the finding of Arnim Weber et al. [20], who reported that most periplasmic proteins in the YceI protein family were induced by high-NaCl stress. We suspected that the deletion of the *preA* gene caused the upregulated expression of associated genes that share metabolic pathways with the periplasmic binding protein in the bacterial response to its dramatically changing external environment; that is, there was some type of compensation mechanism. In addition, we examined several oxidative stress-related genes to further understand the change in the oxidative stress response of the tested strains to changes in oxidative stress levels. All examined genes were upregulated, and peptide methionine oxide reductase *msrA* and catalases *katA* and *katB* showed significantly increased expressions compared with the wild-type strain TH0426, although the increase in the expression of heat shock protein *HSP* was not significant. The loss of high-pH-induced periplasmic proteins in extreme environments may hamper the global regulation of the cells and affect the release of the protective regulatory proteins by the corresponding regulatory factors. Further research is needed to understand the mechanisms that drive the changes observed in this study.

## 4. Materials and Methods

### 4.1. Bacterial Strains, Plasmids and Culture Conditions

The bacterial strains and plasmids used in this study are listed in Table 3. The *A. veronii* TH0426 strain was isolated from the farmed yellow catfish in Zhejiang province; it was identified in our laboratory and deposited in the MCCC (Marine Culture Collection of China) with the deposit number MCCC 1K02718. The complete genome sequence of *A. veronii* TH0426 was deposited in GenBank under accession number CP012504 [21]. The strain was grown in Luria–Bertani (LB) broth or Rimler–Shotts (RS) medium. *Escherichia coli* was cultured in LB liquid medium or plated on LB agar at 37 °C. The vectors pRE112 and pBBR1-MCS were used to recombine with the genomes and express the foreign gene, respectively. When necessary, appropriate concentrations of antibiotics were prepared: 100 μg/mL ampicillin (Amp) and 50 μg/mL chloramphenicol (Cm) were used for the positive selection.

### 4.2. Ethics Statement

Clinically healthy zebrafish used for pathogenicity experiments were purchased from a commercial fish market in Changchun City, China. All the experiments were implemented in strict accordance with the Regulations of Animal Experimentation of Jilin Agricultural University (JLAU08201409), and the animal facility based itself on the National Institutes of Health guide for the care and use of Laboratory animals (NIH Publications No. 8023).

### 4.3. Construction of A. veronii Deletion Mutant (∆preA) and Complemented Strain (C-preA)

The in-frame region of the *preA* gene in the genome of the wild-type *A. veronii* strain TH0426 was deleted by a conjugation reaction according to a previously reported research method [22]. In brief, the flanking regions of the *preA* gene were amplified by PCR with the primers P_1-1_/P_1-2_ (upstream) and P_1-3_/P_1-4_ (downstream). The sequenced and purified fragments were ligated by fusion PCR and cloned into the pEASY plasmid (TransGen Biotech, Beijing, China) skeleton for replication. Then, the fragment was digested by *Sac* I and *Xba* I and attached to the suicide plasmid pRE112, which was treated with the same restriction enzymes and denoted pRE112-*preA*. Recombinant plasmids were introduced into *E. coli* WM3064 to undergo allelic replacement to disrupt the *preA* gene. The first recombination was screened by the LB solid plate containing the appropriate concentrations of Amp and Cm and the second conjugation was carried out to screen for the suspected mutant.

The *preA* gene carrying a promoter region was amplified using the primers P_5-1_/P_5-2_ and P_6-1_/P_6-2_ and ligated into the broad-host-range expressed plasmid pBBR1-MCS to construct the recombinant expression plasmid pBBR-*preA*. The resulting plasmid was then transformed into the mutant strain to construct the complemented strain. Real-time quantitative RT-PCR (qRT-PCR) and DNA sequencing were conducted to identify the mutant strain ∆*preA* and complemented strain C-*preA*. The genetic stabilities of these strains were determined by their continuous subculture results through PCR analysis. The primers used in the construction of ∆*preA* and C-*preA* are listed in Table A1.

### 4.4. Growth Analysis

We referenced previous methods to measure the influence of *preA* on *A. veronii* TH0426 growth [23]. The wild-type strain TH0426, mutant ∆*preA*, and C-*preA* were cultured overnight, and bacterial concentrations were quantified by colony counting. After that, equal volumes of bacterial suspension were placed into new containers with 50 mL of LB and cultured at 28 °C with agitation at 180 rpm. The OD_600_ value of bacterial growth was measured every hour. The experiment was performed three times.

### 4.5. Motility Test

Motility experiments for swimming ability and swarming ability were performed to detect changes in the flagella of the transformed strains (mutant strain ∆*preA* and complemented strain C-*preA*) according to a previous method with some modifications [24]. Briefly, semi-solid LB containing 0.3% agar was prepared to detect swimming ability, and three kinds of bacterial suspension with the same concentration were transplanted into the above medium. Next, the swarming ability of the three strains was detected using the same approach as that in the swimming experiment, with 0.5% agar and 5% glucose added to the medium. All experiments were cultured at 28 °C for over 18 h. Experiments were repeated three times in parallel.

### 4.6. Biofilm Assay

TH0426, ∆*preA,* and C-*preA* were cultured to the exponential phase in LB medium, and then diluted to detect biofilm formation according to a previous method with appropriate modifications [25,26]. The diluted liquid was transferred to a 96-well microplate and incubated at 28 °C for 24 h without shaking. Then, the biofilm formation ability of each strain was determined by measuring the OD_600_ value. The experiment was performed three times.

### 4.7. Pathogenicity Analysis

The median lethal doses (LD_50_) for zebrafish were measured to assess the pathogenicities of the three strains (wild-type *A. veronii*, mutant ∆*preA,* and C-*preA*) according to a previous report [27]. Briefly, after overnight culture, the bacterial concentration was quantified and adjusted so that the three strains had the same concentration. Zebrafish were trained for one week and evenly divided into twenty-four groups with eight fish in each tank. Diluted bacterial suspension was established using eight gradients. Then, a 10 μL volume of bacterial solution was injected intraperitoneally into each fish. The control group was treated with the same volume of PBS instead of the bacterial solution. We then recorded the mortality and removed the fish that died. The LD_50_ value was calculated by Kou’s law.

### 4.8. Adhesion and Invasion Ability

Adhesion and invasion abilities of the strains to EPC cells were detected using a previous method with appropriate modifications [28]. EPC cells (ATCC, DC, US, http://www.atcc.org/) were grown in M199 with 10% heat-inactivated fetal bovine serum and 1% antibiotic (penicillin and streptomycin) at 25 °C in a cell incubator with 5% CO_2_ [29]. After overnight culturing, a single layer EPC cells were counted using a counting chamber. Three strains were also grown and quantified by colony counting. Then, bacteria and EPC cells were mixed thoroughly in a ratio of 10:1 and co-cultured for 1 h in a 25 °C incubator. The control group was treated with an equal volume of sterile PBS. After that, each well was washed three times with PBS, and cells were lysed with 1% TrionX-100 at 25 °C for 45 min. Each well was mixed adequately and diluted by gradient. The number of bacteria adhering to the cell surface was quantified by the plate counting method. Each strain was tested in triplicate, and the adhesion rate was calculated from the mean of three wells. Each experiment was repeated three times.

### 4.9. Cytotoxicity to EPC Cells

The amount of lactate dehydrogenase (LDH) released by the cells was used to evaluate the toxicity of the three strains (parent strain, mutant strain, and complemented strain) according to a previous study [30]. Briefly, the optimal time of cytotoxicity of each strain was determined by observing the status of bacterial infection after 30 min, 1 h, and 2 h, separately. Then, the cytotoxicities of the three strains were detected using the CytoTox 96^®^ Non-Radioactive Cytotoxicity Assay kit. The LDH release percentage was calculated from three replicate experiments.

### 4.10. Stress Test

The stress test measured tolerance to four different stresses: temperature, pH, oxidative stress, and osmotic stress. Stress tolerance was evaluated to investigate the role of the *preA* gene according to previous studies [31,32]. In brief, three strains (TH0426, ∆*preA,* and C-*preA*) were grown to the log phase in LB medium at 28 °C and then harvested by centrifugation. The pellets were washed three times with PBS. For the pH tolerance test, the washed pellets were resuspended in PBS at different pH values (3, 4, 5, 6, 7, 8, 9, and 10) at 28 °C. For the oxidative stress test, the washed pellets were resuspended in PBS containing 1 mM H_2_O_2_ for 1 h at 28 °C. For the temperature stress test, the washed pellets were incubated in PBS for 1 h at 55 °C. For the osmotic tolerance test, the washed bacteria were plated directly on an LB solid plate containing 0.4 M NaCl at 28 °C for 24 h, after which colonies were counted. The pellets were resuspended 1 mL of sterile PBS for 1 h at 28 °C and regarded as blank controls in all tolerance tests. Each tolerance test was repeated in parallel three times.

### 4.11. Expression of Oxidative Stress Related Gene

The reason for changes in oxidative stress tolerance was explored by measuring the mRNA expression of several oxidative stress-related genes following oxidative stress exposure.

Immediately after treatment with hydrogen peroxide, total RNA was extracted from the three strains and reverse-transcribed into complementary DNA (cDNA). Then, qRT-PCR was used to detect the expression of the genes (*katA*, *katB*, *HSP*, *ibpA*, *algW*, and *msrA*) in the pathway of periplasmic protein metabolism and to measure changes in the expression of oxidative stress-related genes in the absence of the *preA* gene. The primers for the oxidative stress-related genes are listed in Table A2.

### 4.12. Statistical Analysis

All data were analyzed by SPSS 16.0 software for one-way ANOVA. GraphPad Prism version 7.0 was used for statistical data. The results of all statistical analyses of the data are represented as the means ± standard deviations (Ms ± SDs). For all tests, statistical significance was defined as *p* < 0.05, *p* < 0.01, or *p* < 0.001.

## 5. Conclusions

Collectively, the results of the present study suggest that the *preA* gene plays an important role in the pathogenicity of *A. veronii* in zebrafish, the oxidative stress resistance of the pathogen, and its toxicity to EPC cells. Disturbance of the *preA* gene’s function severely interfered with oxidative stress-related gene expression. These results provide interesting insights into the role of *preA*-regulated periplasmic binding protein in the pathogenesis of *A. veronii* infection. Therefore, future studies on the function of *preA* will help further elucidate the pathogenic mechanism of *A. veronii*.

## Figures and Tables

**Table 1:** Toxicity to epithelioma papulosum cyprini (EPC) cells.

Strains	Cytotoxicity ^a^ (%)	
	1 h	2 h
TH0426	23.1 ± 0.33	68.3 ± 0.71
∆*preA*	33.4 ± 0.47 *	86.7 ± 0.62 **
C-*preA*	22.5 ± 0.37	60.4 ± 0.42

Data are the means of three experiments and presented as the means ± SDs. ^a^ The amount of lactate dehydrogenase released in the EPC cells. * *p* < 0.05—significant and ** *p* < 0.01—highly significant versus the corresponding values of TH0426.

**Table 2:** Adhesion and invasion ability of *A. veronii* mutant strain ∆*preA* to EPC cells.

Strain	Number of Bacteria ^a^ (CFU/well)	Adhesion and Invasion to EPC (%)
TH0426	(2.4 ± 0.16) × 10^7^	16 ± 1.1
∆*preA*	(4.95 ± 0.05) × 10^7^	33 ± 0.3 *
C-*preA*	(2.25 ± 0.08) × 10^7^	15 ± 0.5

Data are the means of three experiments and presented as means ± SDs. ^a^ Cell counts of bacteria that attach and invade EPC cells. * *p* < 0.05—significant versus the corresponding values of TH0426.

**Table 3:** Bacterial strains and plasmids used in this study. Ampicillin (Amp), chloramphenicol (Cm).

Strains or Plasmids	Description	Source or References
Strains		
*A. veronii* TH0426	Wild-type strain, Amp^r^	This study
∆*preA*	*preA* deletion mutant of TH0426, Amp^r^	This study
C-*preA*	∆*preA* complemented with the whole *preA* gene	This study
*E. coli* Trans1-T1	F- φ80(*lacZ*)ΔM15Δ*lacX*74*hsd*R (rk^−^, mk+) Δ*recA*1398*end*A1*ton*A	TransGene Biotech
*E. coli* DH5α-λpir	λpir lysogen of DH5α	Stored in our lab
*E. coli* WM3064	*thrB1004 pro thi rpsL hsdS lacZ* *∆* *M15RP4-1360(araBAD)567* *∆* *dapA1341: [erm pir(wt)]*	Stored in our lab
Plasmids		
pEASY-Blunt Zero	TA cloning vector, Amp^r^	TransGene Biotech
pEASY-UD *preA*	Carrying a 1015 bp fragment upstream and a downstream 979 bp fragment of the *preA* ORF, Amp^r^	This study
pRE112	pGP704 suicide plasmid, *pir* depengent, *oriT*, *oriV, sacB*, Cm^r^	Stored in our lab
pRE112-UD*preA*	pRE112 carrying a 1015 bp fragment upstream and a downstream 979 bp fragment of the *preA* ORF, Cm^r^	This study
pBBR1-MCS	Broad-host range vector, Cm^r^	Stored in our lab
pBBR-*preA*	pBBR carrying a fragment of 847 bp containing the promoter and *preA* ORF, Cm^r^	This study

## Abbreviations

LB	Luria–Bertani
RS	Rimler–Shotts
EPC	Epithelioma papulosum cyprini
LDH	Lactate dehydrogenase
LD_50_	Median lethal dose
qRT-PCR	Real-time quantitative reverse transcription-polymerase chain reaction
cDNA	Complementary DNA

## Appendix A

**Table A1:** Primers for construction of ∆*preA* and C-*preA*.

Target Gene	Primer	Sequence (5′-3′)
*preA* upstream region	P_1-1_ (*Xba* I)	CGTCTAGAGCCGAGGTGACCGAGGCGACCATGG
	P_1-2_	GGAACTTCTGGCTGCTTCTTTGTACTGATTT
*preA* downstream region	P_1-3_	AAGAAGCAGCCAGAAGTTCCGCGCCCATT
	P_1-4_ (*Sac* I)	TAGAGCTCCCGAGAGCTTGAGGGTGCCGT
*preA* region outside	P_3-1_	GCGCCATGGACGAAGTTGACC
	P_3-2_	ATCCGCAGCGCCAGATCGAA
*preA* ORF internal sequence	P_4-1_	AGGCCACACCCACCACAGGCT
	P_4-2_	CTGCAAACCCTGGCGGCTCC
*preA* promoter sequence	P_5-1_ (*Pst* I)	AACTGCAGTTTCCATCCTCAGTAAATAATGA
	P_5-2_	TCAGTACAAACCGTTCGTTCCTTCATCTT
*preA* ORF sequence	P_6-1_	GAACGAACGGATGGGCGCGGAACTTCTGC
	P_6-2_ (*Hind* III)	GCAAGCTTTCAGTACAAAGAAGCAGCAGGCC
16S rRNA sequence (used for qRT-PCR)	P_7-1_	GCCACGTCTCAAGGACACAG
	P_7-2_	TGGGGAGCAAACAGGATTAGA
*preA* ORF sequence (used for qRT-PCR)	P_8-1_	TCTATGGCATACCGGAAAAGG
	P_8-2_	ACTGAACACCGCAAAGAGCA
pRE112 vector	P_9-1_	GCGATGAGTGGCAGGGC
	P_9-2_	TTACCGACTGCGGCCTGAGT
pBBR1-MCS vector	P_10-1_	TAAGTTGGGTAACGCCAGG
	P_10-2_	GAGTTAGCTCACTCATTAGGC

**Table A2:** Primers for oxidative stress related genes.

Target Gene	Primer	5′-3′Sequence	Band Length (bp)
*msrA*	MsrA-F	GATTTCCACCCTCTTCATGCC	156
	MsrA-R	AGCGGCGACTACCTCTGTGT	
*algW*	AlgW-F	TGCGGGTGTAGATGTTGACG	197
	AlgW-R	GAAAATCCCCCCTTTAGTCAGTTA	
*ibpA*	IbpA-F	GCTTGTTGCCCTTCACCGT	182
	IbpA-R	CGATCGTCTGGCCAATCTCAT	
*katA*	KatA-F	TTCATCTCCCGCAGGCTCTT	195
	KatA-R	CAACACCCCGGTCTTTTTCAT	
*HSP*	HSP-F	TCGGCAATTCCCTGATGG	189
	HSP-R	GAAGACCGCTGCTTACCCC	
*katB*	KatB-F	TGAGCAATAATTCAGACAGTTCCG	143
	KatB-R	CGGATTGGTCTTGGTATCGTGT	

## References

[1] Parte A.C. (2013). LPSN—List of prokaryotic names with standing in nomenclature. Nucleic Acids Res..

[2] Ghenghesh K.S., Abeid S.S., Jaber M.M., Ben-Taher S.A. (1999). Isolation and haemolytic activity of *Aeromonas* species from domestic dogs and cats. Comp. Immunol. Microb..

[3] D’Aloia M.A., Bailey T.A., Samour J.H., Naldo J., Howlett J.C. (1996). Bacterial flora of captive houbara (Chlamydotis undulata), kori (Ardeotis kori) and rufous-crested (Eupodotis ruficrista) bustards. Avian Pathol..

[4] Zhang D.X., Kang Y.H., Song M.F., Shu H.P., Guo S.N., Jia J.P., Tao L.T., Zhao Z.L., Wang C.F., Wang G.Q. (2019). Identity and virulence properties of *Aeromonas* isolates from healthy Northern snakehead (*Channa argus*) in China. Lett. Appl. Microbiol..

[5] Yamada S., Matsushita S., Dejsirilert S., Kudoh Y. (1997). Incidence and clinical symptoms of *Aeromonas*-associated travellers’ diarrhoea in Tokyo. Epidemiol. Infect..

[6] Al Harbi M., Osoba A.O., Mowallad A., Al-Ahmadi K. (2001). Tract microflora in Saudi patients with cholelithiasis. Trop. Med. Int. Health.

[7] Cascon A., Fregeneda J., Aller M., Yugueros J., Temprano A., Hernanz C., Naharro G. (2000). Cloning, characterization, and insertional inactivation of a major extracellular serine protease gene with elastolytic activity from *Aeromonas hydrophila*. J. Fish Dis..

[8] Stancik L.M., Stancik D.M., Schmidt B., Barnhart D.M., Yoncheva Y.N., Slonczewski J.L. (2002). pH-dependent expression of periplasmic proteins and amino acid catabolism in *Escherichia coli*. J. Bacteriol..

[9] Georgellis D., Arvidson S., Gabain A.V. (1992). Decay of ompA mRNA and processing of 9S RNA are immediately affected by shifts in growth rate, but in opposite manners. J. Bacteriol..

[10] Lin J., Smith M.P., Chapin K.C., Baik H.S., Bennett G.N., Foster J.W. (1996). Mechanisms of acid resistance in enterohemorrhagic *Escherichia coli*. Appl. Environ. Microb..

[11] Akashi H., Gojobori T. (2002). Metabolic efficiency and amino acid composition in the proteomes of *Escherichia coli* and *Bacillus subtilis*. Proc. Natl. Acad. Sci. USA.

[12] Handa N., Terada T., Doi-Katayama Y., Hirota H., Tame J.R., Park S.Y., Yokoyama S. (2005). Crystal structure of a novel polyisoprenoid-binding protein from Thermus thermophilus HB8. Protein Sci..

[13] Flower D.R. (1996). The lipocalin protein family: Structure and function. Biochem. J..

[14] Collins M.D., Jones D. (1981). Distribution of isoprenoid quinone structural types in bacteria and their taxonomic implication. Microbiol. Rev..

[15] Terwilliger T.C. (2003). Automated side-chain model building and sequence assignment by template matching. Acta Cryst. D.

[16] Prüβ B.M., Wolfe A.J. (1994). Regulation of acetyl phosphate synthesis and degradation, and the control of flagellar expression in *Escherichia coli*. Mol. Microbiol..

[17] Soballe R., Poolle R.K. (1999). Microbial ubiquinones: Multiple roles in respiration, gene regulation and oxidative stress management. Microbiol..

[18] Daniels D.L., Plunkett G., Burland V., Blattner F.R. (1992). Analysis of the *Escherichia coli* genome: DNA sequence of the region from 84.5 to 86.5 minutes. Science.

[19] Weber A., Kögl S.A., Jung K. (2006). Time-dependent proteome alterations under osmotic stress during aerobic and anaerobic growth in *Escherichia coli*. J. Bacteriol..

[20] Labandeira-Rey M., Brautigam C.A., Hansen E.J. (2010). Characterization of the CpxRA regulon in *Haemophilus ducreyi*. Infect. Immun..

[21] Kang Y.H., Pan X.Y., Xu Y., Siddiqui S.A., Wang C.F., Shan X.F., Qian A.D. (2016). Complete genome sequence of the fish pathogen *Aeromonas veronii* TH0426 with potential application in biosynthesis of pullulanase and chitinase. J. Biotechnol..

[22] Yang B.T., Zhang D.X., Wu T.L., Zhang Z.Q., Raza S.H., Schreurs N., Zhang L., Yang G.L., Wang C.F., Qian A.D. (2019). Maltoporin (LamB protein) contributes to the virulence and adhesion of *Aeromonas veronii* TH0426. J. Fish Dis..

[23] Zhou Z., Pang H., Ding Y., Cai J., Huang Y., Jian J., Wu Z. (2013). VscO, a putative T3SS chaperone escort of *Vibrio alginolyticus*, contributes to virulence in fish and is a target for vaccine development. Fish Shellfish Immunol..

[24] Rashid M.H., Kornberg A. (2000). Inorganic polyphosphate is needed for swimming, swarming, and twitching motilities of *Pseudomonas aeruginosa*. Proc. Natl. Acad. Sci. USA.

[25] Stepanović S., Vuković D., Dakić I., Savić B., Švabić-Vlahović M. (2000). A modified microtiter-plate test for quantification of staphylococcal biofilm formation. J. Microbiol. Methods..

[26] Tu J., Lu F., Miao S., Ni X., Jiang P., Yu H., Hu Q. (2014). The siderophore-interacting protein is involved in iron acquisition and virulence of *Riemerella anatipestifer* strain CH3. Vet. Microbiol..

[27] Schwenteit J., Gram L., Nielsen K.F., Fridjonsson O.H., Bornscheuer U.T., Givskov M., Gudmundsdottir B.K. (2011). Quorum sensing in *Aeromonas salmonicida* subsp. achromogenes and the effect of the autoinducer synthase AsaI on bacterial virulence. Vet. Microbiol..

[28] Mathew J.A., Tan Y.P., Rao P.S.S., Lim T.M., Leung K.Y. (2001). *Edwardsiella tarda* mutants defective in siderophore produciton, motility, serum resistance and catalase activity. Microbiology.

[29] Fijan N., Sulimanović D., Bearzotti M., Muzinić D., Zwillenberg L.O., Chilmonczyk S., De Kinkelin P. (1983). Some properties of the epithelioma papulosum cyprini (EPC) cell line from carp Cyprinus carpio. Ann. Inst. Pasteur/Virol..

[30] Stintzi A., Raymond K.N. (2000). Amonabactin-mediated iron acquisition from transferrin and lactoferrin by *Aeromonas hydrophila*: Direct measurement of individual microscopic rate constants. J. Biol. Inorg. Chem..

[31] Lv Y., Xiao J., Liu Q., Wu H., Zhang Y., Wang Q. (2012). Systematic mutation analysis of two-component signal transduction systems reveals EsrA-EsrB and PhoP-PhoQ as the major virulence regulators in *Edwardsiella tarda*. Vet. Microbiol..

[32] Li Q., Zhang Y., Dechao D., Yanfei Y., Zhang W. (2018). Characterization and functional analysis of PnuC that is involved in the oxidative stress tolerance and virulence of *Streptococcus suis* serotype 2. Vet. Microbiol..

